# Development of a novel diagnostic model for Alzheimer’s disease based on glymphatic system and metabolism-related genes

**DOI:** 10.3389/fmolb.2025.1585761

**Published:** 2025-12-05

**Authors:** Ailing Jiang, Danli Shi, Xianting Que, Ziqun Lin, Yanlan Chen, Yanzhen Huang, Chao Liu, Yishuang Wen, Shuyi Zhang, Wen Huang

**Affiliations:** 1 Department of Neurology, The First Affiliated Hospital of Guangxi Medical University, Nanning, Guangxi, China; 2 Department of Neurology, Jiangbin Hospital, Nanning, Guangxi, China

**Keywords:** Alzheimer’s disease, glymphatic system, metabolism-related genes, diagnostic model, biomarkers

## Abstract

**Objectives:**

Alzheimer’s disease (AD), a common neurodegenerative disorder, is characterized by its complex pathogenesis and challenging early diagnosis; however, the role of the glymphatic system and metabolism-related genes (GS&MetabolismRGs) in AD remains poorly understood. Therefore, this study aimed to explore a potential diagnostic model and the molecular mechanisms of GS&MetabolismRGs in AD.

**Materials and methods:**

We obtained glymphatic system and metabolism-related differentially expressed genes (GS&MetabolismRDEGs) associated with AD by integrating of GEO and GeneCards databases. Gene Ontology analysis, Kyoto Encyclopedia of Genes and Genomes enrichment analyses, and gene set enrichment analysis were performed to investigate the roles of GS&metabolismRDEGs in AD-related biological processes. Hub genes were identified using machine learning methods, resulting in the construction and validation of AD diagnostic models. AD samples were further stratified into high-score and low-score groups based on the median value of glymphatic system and Metabolism Score to investigate the underlying pathogenesis. Finally, immune infiltration analysis was conducted to explore the relationship between immune cell frequencies and hub genes.

**Results:**

Six GS&MetabolismRDEGs were identified, which were predominantly enriched in biological processes, such as the PD-L1 expression, hyaluronan metabolic process, and the PD-1 checkpoint pathway in cancer. Further analysis identified six hub genes that were used to construct an AD diagnostic model. Immune infiltration analysis of the disease and control groups revealed significant associations among all eight immune cell types. The strongest negative correlation was found between the resting memory CD4^+^ T cells and Tregs. Further analysis revealed a strong positive correlation between Tregs and *NFKB1* in low-risk group and the most significant correlation between activated mast cells and *TREM1* in high-risk group.

**Conclusion:**

This study developed a novel diagnostic model based on six GS&MetabolismRDEGs, highlighting their potential as key biomarkers for early diagnosis and providing new insights into the molecular mechanisms driving AD.

## 1 Introduction

Alzheimer’s disease (AD) is a common neurodegenerative disorder characterized by progressive cognitive decline across multiple domains, psychiatric and behavioral changes, and loss of daily living abilities at its advanced stages (Gustavsson et al., 2023). Owing to an increase in the aging population, the incidence of AD has increased, with AD consequently becoming a significant public health concern. By 2023, more than 50 million people worldwide are estimated to be affected by AD, with the number expected to reach 150 million by 2050. The annual economic burden of AD exceeds one trillion dollars, imposing a heavy burden on both society and families (Gustavsson et al., 2023). Current therapeutic strategies for AD primarily involve pharmacological interventions and cognitive behavioral therapies. Although these treatments may achieve modest improvements in cognitive function, they are insufficient to halt or reverse disease progression (Shi et al., 2022; Cummings et al., 2023). The disease is often diagnosed in its advanced stages, owing to the lack of reliable preclinical biomarkers. Thus, identifying early diagnostic biomarkers and understanding their underlying molecular mechanisms are crucial for disease management and personalized therapy.

Accumulation of amyloid-beta (Aβ) and tau proteins, partly because of impaired protein clearance mechanisms, is a pathological hallmark of AD (Da Mesquita et al., 2018; Jack et al., 2018). Recent studies have highlighted the pivotal role of glymphatic system (GS) dysfunction in AD. GS, which clears Aβ and tau proteins, operates via the exchange of cerebrospinal and interstitial fluid via aquaporin-4 (AQP4) located on astrocytic endfeet. Accumulating evidence indicates that GS dysfunction promotes amyloid-β and tau accumulation, disrupts cerebral metabolic homeostasis and evokes oxidative stress, synaptic failure and neuroinflammation, thereby accelerating neuronal loss and cognitive decline (Mestre et al., 2020). Impaired GS also disrupts cellular energy metabolism, induces oxidative stress, and negatively affects synaptic function, thereby accelerating tau hyperphosphorylation and promoting neuronal death (Doroszkiewicz et al., 2024; Perluigi et al., 2024). Metabolic derangements, in turn, impair glymphatic clearance, with both processes interconnected through gene regulatory and immune networks to drive AD pathogenesis. Contemporary diagnostic approaches for AD primarily focus on amyloid-β and tau pathologies, limiting mechanistic insights and hindering clinical translation. These unidimensional frameworks fail to capture the multifaceted nature of AD, particularly the GS’s pivotal role in maintaining cerebral metabolic homeostasis. A diagnostic model that captures these glymphatic–metabolic interactions is likely to reflect the disease’s multifactorial pathology more accurately than traditional single-pathway approaches, thereby offering a robust foundation for early diagnosis as well as personalized intervention. Several studies have suggested a significant correlation between altered metabolic states and GS dysfunction (Tian et al., 2024). The interaction between metabolic dysregulation and immune system activation, particularly microglia and T cells, plays a crucial role in AD pathogenesis by promoting chronic neuroinflammation and further exacerbating the pathological cycle of Aβ accumulation and tau hyperphosphorylation (Hasel et al., 2023; Qiu et al., 2022; Wang et al., 2023; Yin et al., 2016). GS-related gene expression profiles in mice with disrupted meningeal lymphatic function differ significantly from those in controls (Da Mesquita et al., 2021). However, although the involvement of the GS in AD is well established, its coordinated regulation with metabolism-related genes and its potential as composite biomarker remain poorly understood (Astara et al., 2024; Hu et al., 2024).

In this study, we present the first integrated analysis of glymphatic system- and metabolism-related differentially expressed genes (GS&MetabolismRDEGs) within a glymphatic-metabolic-immune network, systematically uncovering novel interactions and functional roles. By integrating multi-omics data, functional enrichment, and machine learning, we constructed and validated a diagnostic model for AD based on GS&MetabolismRDEGs. This framework expands the molecular diagnostics paradigm and establishes a foundation for early diagnosis. Therefore, these findings not only address gaps in the current literature but also hold promise for identifying novel biomarkers and developing personalized therapeutic strategies for AD.

## 2 Materials and methods

### 2.1 Data download

The AD datasets GSE63060 (Sood et al., 2015) and GSE63061 (Sood et al., 2015) were obtained from the Gene Expression Omnibus (GEO) database (Barrett et al., 2013) (https://www.ncbi.nlm.nih.gov/geo/) using the R package, GEOquery (Davis and Meltzer, 2007). The datasets were batch-corrected using the R package sva (Leek et al., 2012), resulting in a combined GEO dataset (combined dataset) as the training set. A similar approach was applied to download the GSE97760 (Naughton et al., 2015) dataset, which served as the validation set. The data were normalized and standardized using the limma package in R (Ritchie et al., 2015), with probe annotations and normalization procedures applied. Principal component analysis (PCA) (Kamel Ben Salem, 2021) was applied to the expression matrices prior to and following batch correction to assess the effectiveness of batch effect removal.

GS&MetabolismRGs were retrieved from the GeneCards database (Stelzer et al., 2016) (https://www.genecards.org/).

### 2.2 Identification of GS&MetabolismRDEGs and function enrichment analysis

Differentially Expresse Genes (DEGs) between AD samples and control samples were identified across the combined datasets using the limma package in R. The intersection of DEGs with GS&MetabolismRGs was determined, yielding a set of GS&MetabolismRDEGs. Gene Ontology (GO) and Kyoto Encyclopedia of Genes and Genomes (KEGG) enrichment analyses of GS&MetabolismRDEGs were conducted using the R package clusterProfiler (Yu et al., 2012). KEGG pathway visualization was subsequently performed with the R package Pathview (Luo and Brouwer, 2013).

### 2.3 Gene set enrichment analysis (GSEA) of the disease and control group

Genes in the combined datasets were first ranked based on their logFC values comparing the AD group to the control group. Subsequently, GSEA was conducted using the clusterProfiler package in R, encompassing all genes within the combined datasets. The gene sets c2.all.v2023.2.Hs.symbols were retrieved from the Molecular Signatures Database (MSigDB).

### 2.4 Construction of the AD diagnostic model

Logistic regression analysis was conducted on the GS&MetabolismRDEGs to develop an AD diagnostic model from the combined datasets. The expression patterns of GS&MetabolismRDEGs included in the model were visualized using a forest plot. To further refine the GS&MetabolismRDEG selection, a support vector machine (SVM) model (Sanz et al., 2018) was constructed. Least absolute shrinkage and selection operator (LASSO) regression analysis was performed to build an AD diagnostic model using the R package glmnet (Engebretsen and Bohlin, 2019), with the selected GS&MetabolismRDEGs serving as hub genes (core GS&MetabolismRDEGs). The LASSO risk score (RiskScore) was computed using the coefficients obtained from the LASSO regression, with the following formula:

$$RiskScore=\sum_{i}^{\;}Coefficient\;\left({gene}_{i}\right)*mRNA\;Expression\;\left({gene}_{i}\right)$$

### 2.5 Validation of the AD diagnostic model

A nomogram was constructed to visualize the interrelationships among the different model genes using the R package rms. Calibration curves were generated to assess the accuracy and discriminative ability of the AD diagnostic model. Decision curve analysis (DCA) was conducted using the R package ggDCA (Van Calster et al., 2018) to evaluate the clinical utility of the model. Additionally, receiver operating characteristic (ROC) curves were generated using the pROC package (Robin et al., 2011) based on the RiskScore, and the area under the curve (AUC) was determined to assess the diagnostic accuracy. Finally, functional similarity analysis (friends) was carried out to examine the functional relationships among core GS&MetabolismRDEGs using the R package GOSemSim (Yu et al., 2010).

### 2.6 Construction high-score and low-score groups

The glymphatic system and metabolism scores (GS&Metabolism scores) were computed using the single-sample Gene-Set Enrichment Analysis (ssGSEA) algorithm, as implemented in the GSVA package in R. The AD samples were stratified into high-score (HighScore) and low-score (LowScore) groups according to the median value of the GS&Metabolism score. A comparative analysis was conducted to further investigate the expression variation of core GS&MetabolismRDEGs in AD samples between HighScore and LowScore groups. To assess the diagnostic potential of the core GS&MetabolismRDEGs in AD, ROC curves were plotted, and the AUC was calculated.

### 2.7 Highscore and lowScores subgroup GSEA

Differential expression analysis was further performed to compare the HighScore with the LowScore groups. Genes in the combined datasets were sorted according to the logFC values between the HighScore and LowScores groups, followed by GSEA using the clusterProfiler package. The gene sets c2.all.v2023.2.Hs.symbols employed in GSEA were retrieved from MSigDB.

### 2.8 Construction of regulatory networks

Transcription factors (TFs)-regulating core GS&MetabolismRDEGs were retrieved from the ChIPBase database (http://rna.sysu.edu.cn/chipbase/) (Zhou et al., 2017). Additionally, microRNAs (miRNAs) linked to the core GS&MetabolismRDEGs were retrieved from the StarBase v3.0 database (https://starbase.sysu.edu.cn/) (Holliday et al., 2017). The mRNA-TF-miRNA regulatory network was visualized by Cytoscape (Shannon et al., 2003).

### 2.9 Validation and ROC curve analysis of core GS&MetabolismRDEGs

To explore the expression differences of core GS&MetabolismRDEGs between AD samples and control samples in both the combined datasets and the validation GSE97760 dataset, comparison plots were generated based on the expression levels of these core GS&MetabolismRDEGs. The pROC package was utilized to generate ROC curves and calculate the AUC to validate the diagnostic efficacy of core GS&MetabolismRDEGs in AD.

### 2.10 Immune infiltration analysis (CIBERSORT)

Immune cell infiltration profiles in the combined datasets were generated using the CIBERSORT algorithm (Newman et al., 2015) by incorporating the immune cell signature gene matrix. The differences in immune cell expression between the AD samples and control samples was visualized using the ggplot2 package. Spearman’s algorithm was applied to assess the association between core GS&MetabolismRDEGs and immune cell types. A similar analysis was performed to evaluate immune cell infiltration in the high-risk (HighRisk) and low-risk (LowRisk) groups.

### 2.11 Statistical analysis

All data processing and analyses were conducted using R software (version 4.4.1). Statistical comparisons between the two groups for continuous variables were performed using the independent Student’s t-test (for normally distributed data) or the Mann–Whitney U test (for non-normally distributed data), unless otherwise specified. Comparisons between three or more groups were performed using the Kruskal–Wallis test. Spearman’s correlation analysis was used to compute the correlation coefficients between different molecular features. All statistical tests were two-sided, with statistical significance set at p < 0.05.

## 3 Results

### 3.1 Technology roadmap

**FIGURE 1:**
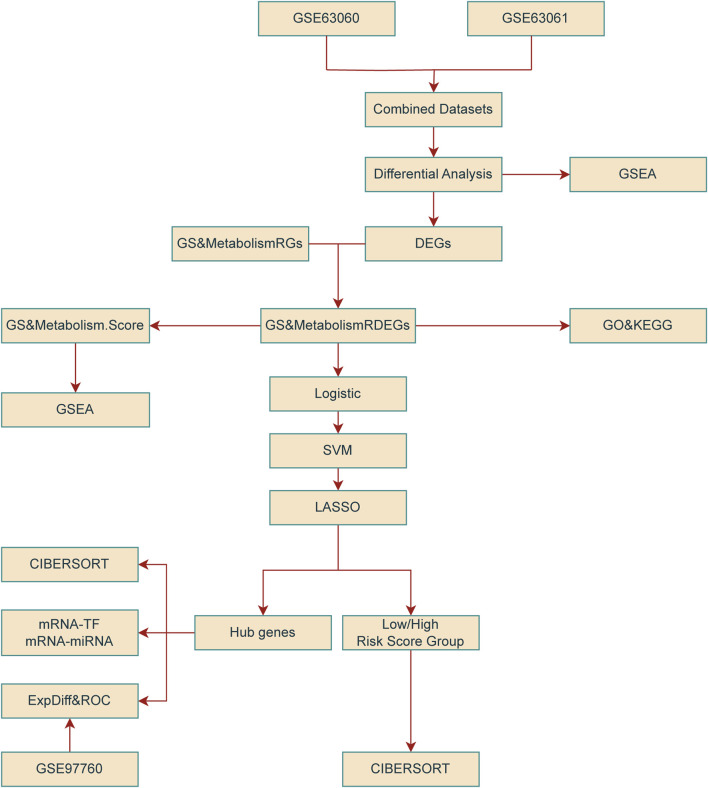
Technology roadmap. DEG, differentially expressed genes; ExpDiff and ROC, expression difference and receiver operating characteristic; GO, Gene Ontology; GS&MetabolismRDEG, glymphatic system and metabolism-related differentially expressed gene; GS&MetabolismRG, glymphatic system and metabolism-related gene; GSEA, gene-set enrichment analysis; KEGG, Kyoto Encyclopedia of Genes and Genomes; LASSO, least absolute shrinkage and selection operator; SVM, support vector machine; TF, transcription factor.

**FIGURE 2:**
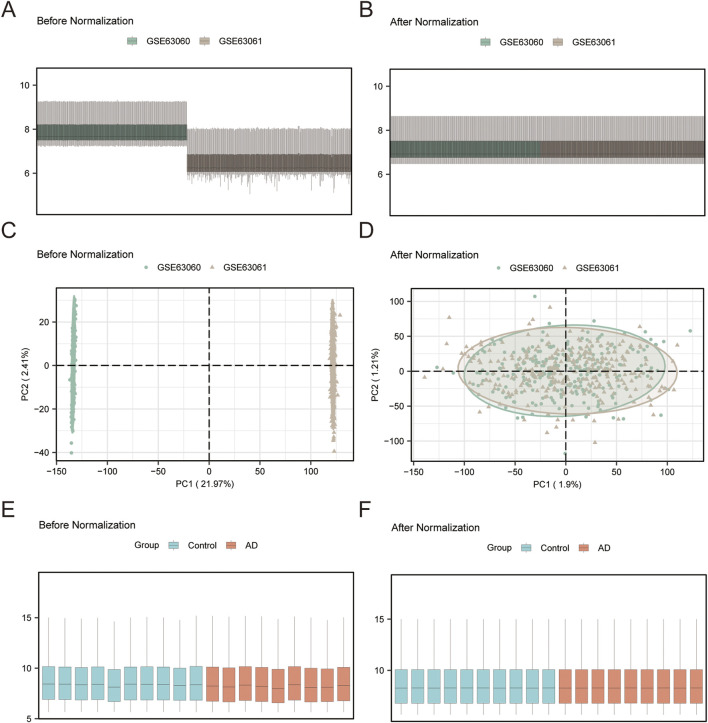
Dataset processing of combined datasets and GSE97760 **(A)** Distribution boxplot of the GEO dataset (combined datasets) before batch processing. **(B)** Boxplot of the distribution of combined datasets after batch processing. **(C)** PCA plot of the dataset before batch processing. **(D)** PCA plot of the integrated GEO dataset (combined datasets) after batch processing. **(E)** Distribution boxplot of the GSE97760 dataset before normalization. **(F)** Distribution boxplot of the GSE97760 dataset after normalization. AD, Alzheimer’s disease; PCA, principal component analysis. Green indicates AD dataset GSE63060, brown indicates AD dataset GSE63061, blue indicates the control sample, and orange indicates the AD sample.

Figure 1 illustrates the technical framework of this study. The combined datasets consisted of 284 AD samples and 238 control samples. The validation set comprised 9 AD samples and 10 control samples. GS&MetabolismRGs are presented in Supplementary Tables S1, S2. After removing duplicates, 18 GS&MetabolismRGs were identified in Supplementary Table S3. Following batch effect correction, the batch effects in the combined datasets were notably minimized (Figures 2A–D). The distribution box plots demonstrated consistent expression patterns among the samples in the GSE97760 dataset after data processing (Figures 2E,F).

### 3.2 Differentially expressed GS&MetabolismRGs in AD

**FIGURE 3:**
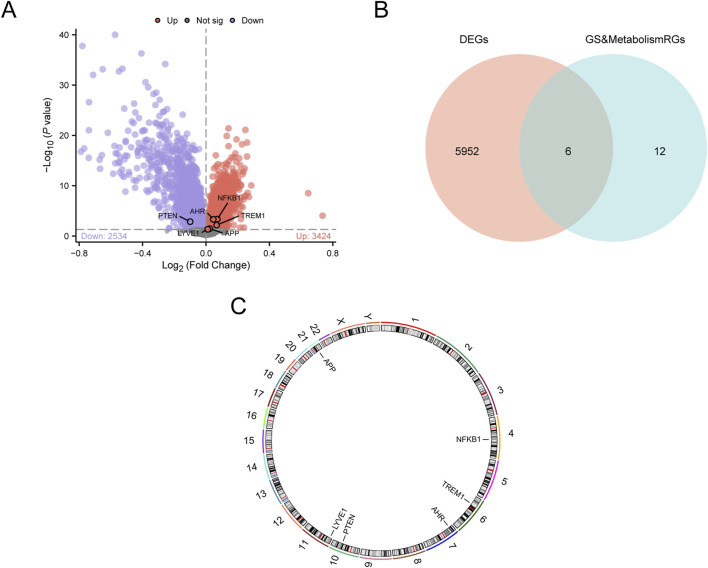
Differential gene expression analysis **(A)** Volcano plot of differentially expressed genes between AD and control samples from the combined GEO datasets. **(B)** Venn diagram of DEGs and GS&MetabolismRGs in the integrated GEO dataset (combined datasets). **(C)** Chromosome localization map of GS&MetabolismRDEGs. AD, Alzheimer’s disease; DEG, differentially expressed gene; GEO, Gene Expression Omnibus; GS&MetabolismRG, glymphatic system and metabolism-related gene.

A total of 5,958 DEGs were identified between the AD samples and control samples, including 3,424 upregulated and 2,534 downregulated genes (Figure 3A). To identify GS&MetabolismRDEGs, the DEGs were intersected with GS&MetabolismRGs, and the overlap was visualized using a Venn diagram (Figure 3B). Overall, six GS&MetabolismRDEGs, *NFKB1*, *AHR*, *PTEN*, *TREM1*, *APP*, and *LYVE1*, were identified. The chromosomal locations of these genes were determined using the R package RCircos, and their positions were mapped onto the chromosomal map shown in Figure 3C. Mapping revealed the following chromosomal locations: *NFKB1* on chromosome 4, *TREM1* on chromosome 6, *AHR* on chromosome 7, *PTEN* on chromosome 10, *LYVE1* on chromosome 11, and *APP* on chromosome 21.

### 3.3 Functional enrichment analysis

**FIGURE 4:**
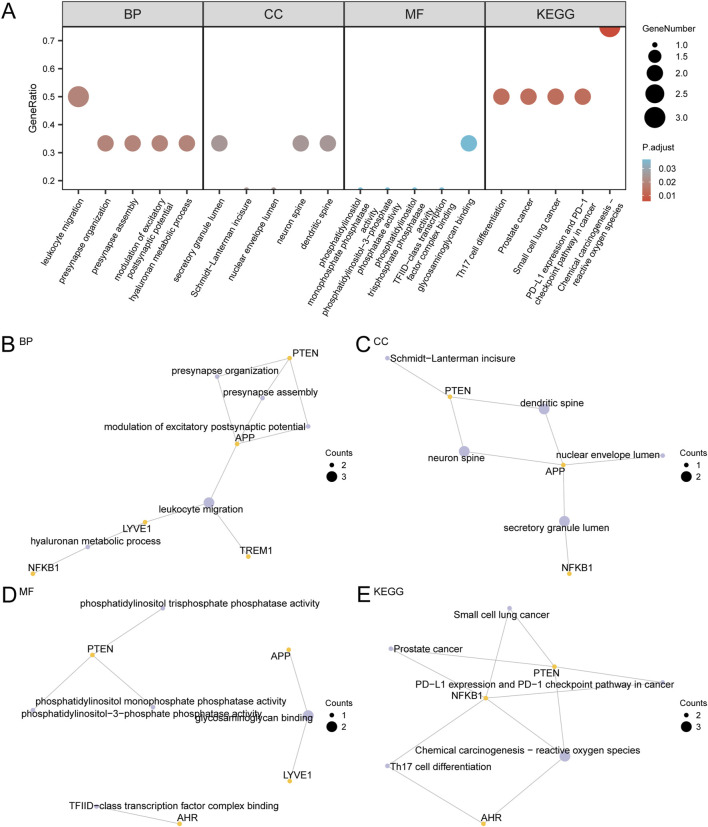
Results of GO and KEGG enrichment analysis for GS&MetabolismRDEGs. **(A)** The results of GO and KEGG enrichment analysis for GS&MetabolismRDEGs, presented as a bubble diagram for BP, CC, MF, and biological pathways (KEGG). The abscissa represents GO and KEGG terms. **(B–E)**. GO and KEGG enrichment analysis results for GS&MetabolismRDEGs are shown in network maps: BP **(B)**, CC **(C)**, MF **(D)**, and KEGG **(E)**. Purple nodes represent entries, yellow nodes represent molecules, and lines represent the relationships between entries and molecules. GS&MetabolismRDEG, glymphatic system and metabolism-related differentially expressed gene; GO, Gene Ontology; KEGG, Kyoto Encyclopedia of Genes and Genomes; BH, Benjamini–Hochberg; BP, biological process; CC, cellular component; MF, molecular function. In the bubble diagram, the size of the bubble represents the number of genes, and the color of the bubble represents the adj.p-value. Redder colors indicate smaller adj.p-values, and bluer colors indicate larger adj.p-values. The screening criteria for GO and KEGG enrichment analysis were adj. p-value <0.05 and FDR value (q value) <0.25, with p-value correction using BH.

Functional enrichment analyses were performed to gain a deeper understanding of the biological processes (BP), cellular components (CC), molecular functions (MF), and biological pathways related to the six GS&MetabolismRDEGs in AD. The results revealed a significant enrichment of these genes in multiple BPs, including hyaluronan metabolic processes, modulation of excitatory postsynaptic potential, presynaptic assembly, presynapse organization, and leukocyte migration. At the cellular level, these genes were predominantly localized in components, such as the dendritic spine, neuronal spine, nuclear envelope lumen, Schmidt-Lanterman incisure, and secretory granule lumen. MFs primarily included glycosaminoglycan binding, TFIID-class transcription factor complex binding, and various phosphatase activities, including phosphatidylinositol trisphosphate phosphatase, phosphatidylinositol-3-phosphate phosphatase, and phosphatidylinositol monophosphate phosphatase activities. Furthermore, these GS&MetabolismRDEGs were significantly enriched in multiple KEGG pathways, including the PD-1 checkpoint pathway, small cell lung cancer, chemical carcinogenesis via reactive oxygen species, PD-L1 expression, prostate cancer, and Th17 cell differentiation (Figures 4A–E).

### 3.4 Construction of the AD diagnostic model

**FIGURE 5:**
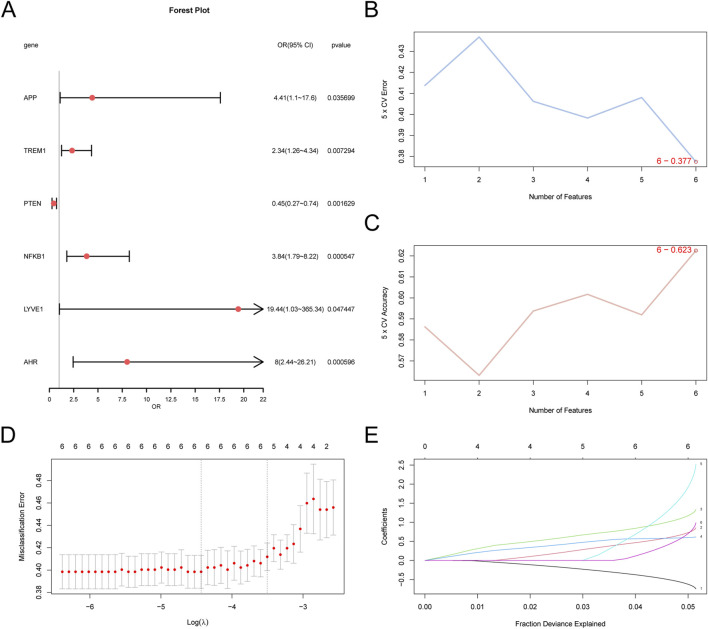
Diagnostic model of AD **(A)** Forest plot of the six GS&MetabolismRDEGs included in the logistic regression model used in the AD diagnostic model. **(B)** The number of genes with the lowest error rate. **(C)** The number of genes with the highest accuracy, obtained using **(B,C)**. Visualization of the SVM algorithm. **(D)** Diagram of the diagnostic model. **(E)** Variable locus diagram of **(D,E)**. LASSO regression model. AD, Alzheimer’s disease; GS&MetabolismRDEG, glymphatic system and metabolism-related differentially expressed gene; LASSO, least absolute shrinkage and selection operator; SVM, support vector machine.

To evaluate the diagnostic potential of the six GS&MetabolismRDEGs in AD, we performed logistic regression analyses on these genes and developed a logistic regression mode (Figure 5A). All six GS&MetabolismRDEGs (*NFKB1*, *AHR*, *PTEN*, *TREM1*, *APP*, and *LYVE1*) showed significant associations (p < 0.05). An SVM model incorporating these six genes was constructed, and it showed that the model achieved the lowest error rate (Figure 5B) and the highest accuracy (Figure 5C) when all six genes were included. To further refine the AD diagnostic model, LASSO regression was performed, which confirmed that *NFKB1*, *AHR*, *PTEN*, *TREM1*, *APP*, and *LYVE1* were core GS&MetabolismRDEGs in the model (Figures 5D,E).

### 3.5 Validation of the AD diagnostic model

**FIGURE 6:**
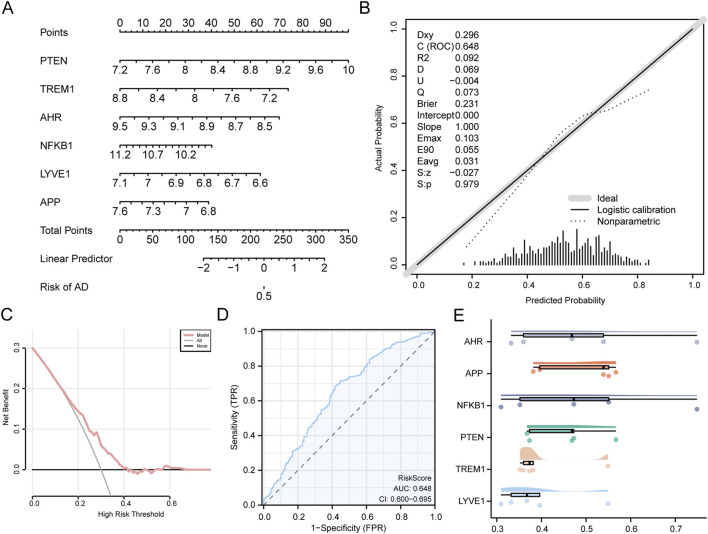
Diagnostic and validation analysis of AD **(A)** Nomograms of hub genes in the combined GEO datasets for the AD diagnostic model. **(B,C)**. Diagnostic model for AD, based on the calibration **(B)** and DCA **(C)** plots of the hub genes in the integrated GEO dataset (combined datasets). **(D)** RiskScore ROC curve in the integrated GEO dataset (combined datasets). **(E)** Functional similarity (friends) analysis results of hub genes presented in the cloud-rain map. The vertical coordinate of the DCA plot represents net income, while the horizontal coordinate represents the probability threshold or threshold probability. AD, Alzheimer’s disease; AUC, area under the curve; DCA, decision curve analysis; FPR, false-positive rate; GEO, Gene Expression Omnibus; ROC, receiver operating characteristic; TPR, true positive rate; AUC >0.5 indicates a tendency for the expression of molecules to promote event occurrence, with the closer AUC is to 1, the better the diagnostic effect. The accuracy of AUC is considered low at 0.5–0.7, moderate at 0.7–0.9, and high at >0.9.

To further evaluate the diagnostic potential of the model, a nomogram based on six core GS&MetabolismRDEGs was constructed (Figure 6A). The findings demonstrated that *PTEN* expression had a markedly higher diagnostic value than the other variables. Calibration analysis was conducted to assess the accuracy and discriminative power of the AD diagnostic model, which demonstrated that the calibration line (dashed line) was closely aligned with the ideal diagonal, suggesting a strong agreement between the observed and predicted values (Figure 6B). The DCA results further support the robustness of the model, showing a substantial net benefit (Figure 6C). Additionally, the ROC curve analysis (Figure 6D) indicated an AUC value for the RiskScore in the combined datasets ranging between 0.5 and 0.7, reflecting moderate diagnostic performance. The formula for calculating the RiskScore was as follows:

$$RiskScore=PTEN\times \left(-0.5944\right)+TREM1\times \left(0.6983\right)+AHR\times \left(1.1344\right)+NFKB1\times \left(0.5960\right)+LYVE1\times \left(1.7754\right)+APP\times \left(0.6359\right).$$

Friend analysis revealed that *AHR* exhibited the strongest correlation with other core GS&MetabolismRDEGs, indicating a pivotal role of *AHR* in the pathophysiology of AD (Figure 6E).

### 3.6 HighScore and lowScore groups analysis

**FIGURE 7:**
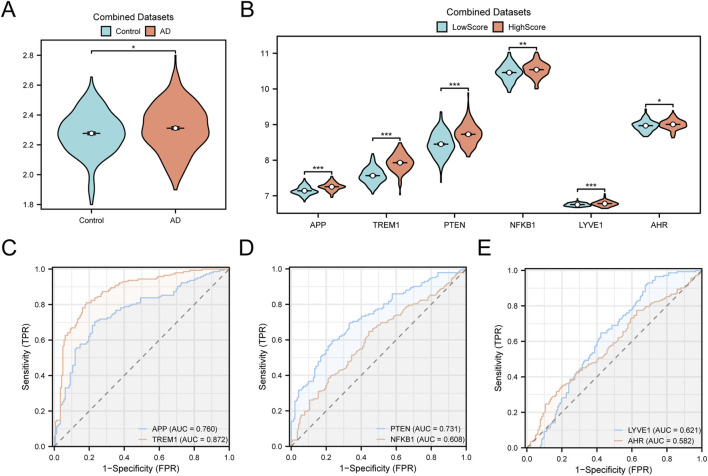
ssGSEA score analysis **(A)** Comparison of the GS&Metabolism score between the control and AD groups in the combined GEO datasets. **(B)** Grouping comparison of hub genes between the HighScore and LowScore groups in AD samples. **(C–E)**. ROC curves of six hub genes: *APP* and *TREM1* **(C)**, *PTEN* and *NFKB1* **(D)**, *LYVE1* and *AHR* **(E)** in AD samples. AD, Alzheimer’s disease; AUC, area under the curve; GS&Metabolism score, glymphatic system and metabolism score; ROC, receiver operating characteristic; ssGSEA, single-sample gene-set enrichment analysis. *p-value <0.05 is statistically significant; **p-value <0.01 is highly statistically significant; ***p-value <0.001 is extremely statistically significant. An AUC >0.5 indicates that molecule expression tends to promote event occurrence; the closer AUC is to 1, the better the diagnostic effect. The accuracy of AUC is considered low at 0.5–0.7, moderate at 0.7–0.9, and high at >0.9. Blue represents control and LowScore; orange represents AD and HighScore.

The GS&Metabolism score exhibited statistically significant differences between AD and control groups (Figure 7A, p < 0.05). The expression levels of the six core GS&MetabolismRDEGs exhibited significant differences between the HighScore and LowScore groups (Figure 7B, p < 0.05). Specifically, *APP*, *TREM1*, *PTEN*, and *LYVE1* showed extremely significant differences (p < 0.001), *NFKB1* was highly significant (p < 0.01), and *AHR* was statistically significant (p < 0.05). ROC curve analysis of the six core GS&MetabolismRDEGs in the AD samples indicated that *APP*, *TREM1*, and *PTEN* exhibited excellent diagnostic performance, while *NFKB1*, *LYVE1*, and *AHR* showed moderate diagnostic accuracy (Figures 7C–E).

### 3.7 GSEA of the highScore and lowScore groups

**FIGURE 8:**
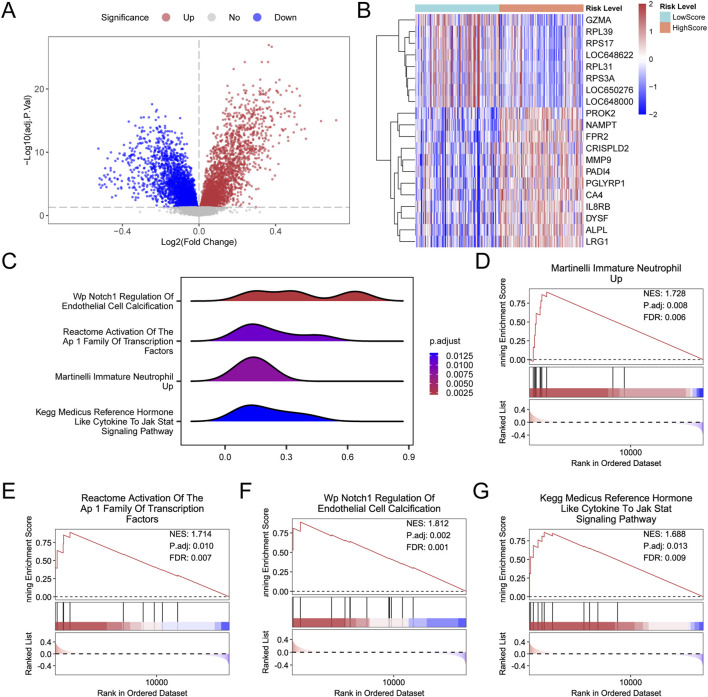
Differential gene expression analysis and GSEA for combined datasets **(A,B)**. Volcano plot **(A)** and heatmap of expression values **(B)** in the differential gene expression analysis for the HighScore and LowScore groups in the combined GEO dataset. **(C)** Four biological function mountain maps of GSEA in the integrated GEO dataset (combined datasets). **(D–G)**. GSEA showing significant enrichment in the integrated GEO dataset (combined datasets) for Martinelli Immature Neutrophil Up **(D)**, Activation of The AP 1 Family of Transcription Factors **(E)**, Notch1 Regulation of Endothelial Cell Calcification **(F)**, Medicus Reference Hormone-Like Cytokine to JAK-STAT Signaling Pathway **(G)**. AD, Alzheimer’s disease; BH, Benjamini–Hochberg; GEO, Gene Expression Omnibus; GSEA, gene-set enrichment analysis. Orange represents the HighScore group, and blue represents the LowScore group. In the heatmap, red indicates high expression and blue indicates low expression. The color in the mountain map represents the adj.p-value; redder colors indicate smaller adj.p-values, and bluer colors indicate larger adj.p-values. The screening criteria for GSEA were adj.p-value <0.05 and FDR value (q value) <0.25, with p-value correction using BH.

A total of 6,192 DEGs were identified between HighScore and LowScore groups, comprising 2,680 upregulated and 3,512 downregulated genes (Figure 8A). The top 20 DEGs were selected for further analysis (Figure 8B). GSEA revealed significant enrichment in biological functions and signaling pathways (Figure 8C), including “Martinelli Immature Neutrophil Up” (Figure 8D), “Activation of the AP1 Family of Transcription Factors” (Figure 8E), “Notch1 Regulation of Endothelial Cell Calcification” (Figure 8F), and “Medicus Reference Hormone-like Cytokine to JAK-STAT Signaling Pathway” (Figure 8G).

### 3.8 Construction of regulatory networks

**FIGURE 9:**
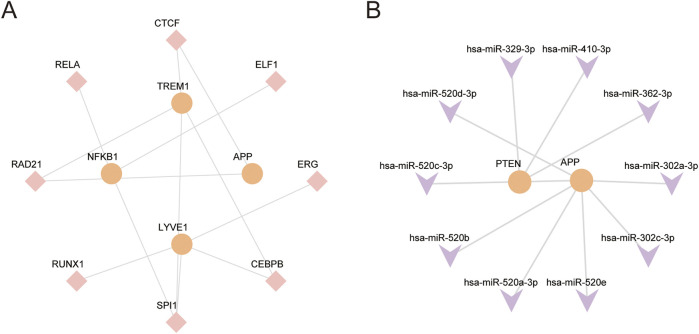
Regulatory network of hub genes **(A)**. The mRNA-TF regulatory network of hub genes. **(B)** The mRNA-miRNA regulatory network of hub genes. RBP, RNA-binding protein; TF, transcription factor. Orange represents mRNA, pink represents TF, and purple represents miRNA.

The TFs and miRNA associated with core GS&MetabolismRGs were initially determined using the ChIPBase database. The results revealed that *TREM1, APP, NFKB1*, and *LYVE1*, are regulated by 8 TFs. Additionally, *PTEN* and *APP* are regulated by 10 miRNAs (Figures 9A,B).

### 3.9 Validation and ROC curve analysis of core GS&MetabolismRGs

**FIGURE 10:**
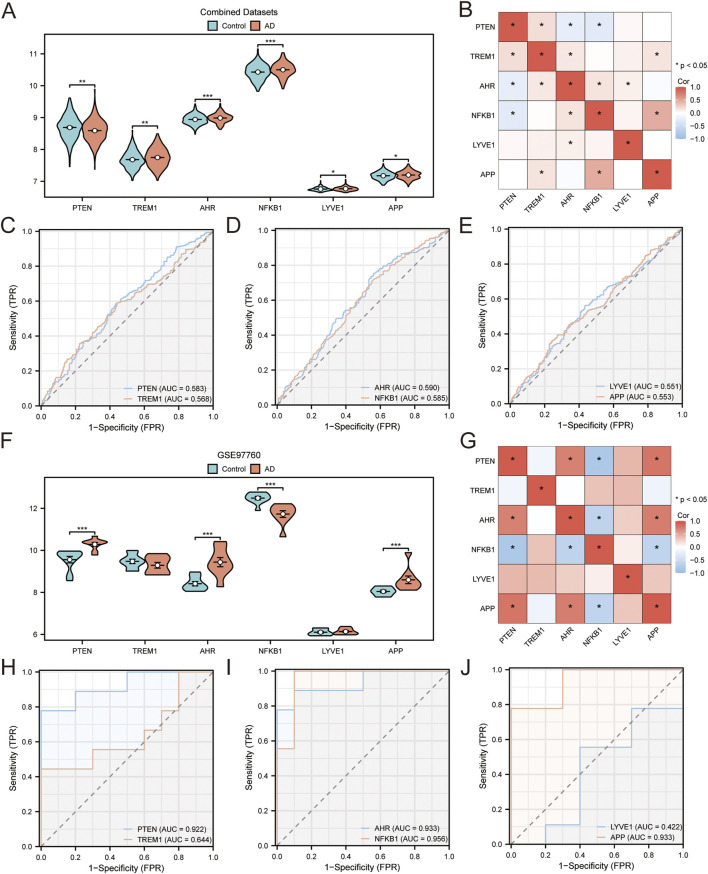
Differential expression validation and ROC curve analysis **(A)**. Grouping comparison of hub genes in AD and control samples in the combined GEO dataset. **(B)** Heatmap of the correlations between hub genes in the combined datasets. **(C–E)**. ROC curves for hub genes: *PTEN* and *TREM1* **(C)**, *AHR* and *NFKB1* **(D)**, and *LYVE1* and *APP* **(E)** in the integrated GEO dataset. **(F)** Group comparison plot of hub genes in AD and control samples in the GSE97760 dataset. **(G)** Heatmap of correlations between hub genes in the GSE97760 dataset. **(H–J)** ROC curves for hub genes: *PTEN* and *TREM1* **(C)**, *AHR* and *NFKB1* **(D)**, and *LYVE1* and *APP* **(E)** in the GSE97760 dataset. p-value <0.05 is statistically significant; ** p-value <0.01 is highly statistically significant; *** p-value <0.001 is extremely statistically significant. AUC >0.5 indicates that molecule expression tends to promote event occurrence; the closer the AUC is to 1, the better the diagnostic effect. The accuracy of AUC is considered low at 0.5–0.7, moderate at 0.7–0.9, and high at >0.9. Blue represents control samples and orange represents AD samples.

The expression levels of the six core GS&MetabolismRGs were assessed in both AD samples and control samples across the combined datasets. The findings revealed that *AHR* and *NFKB1* expression levels were extremely significant differences (p < 0.001). *PTEN* and *TREM1* showed highly significant differences (p < 0.01), whereas *LYVE1* and *APP* showed statistically significant differences in expression levels (Figure 10A, p < 0.05). Correlation analysis revealed that *APP* and *NFKB1* exhibited the strongest positive correlation (Figure 10B, r = 0.46, p < 0.05). ROC curve analysis indicated that *NFKB1*, *AHR*, *PTEN*, *TREM1*, *APP*, and *LYVE1* expression levels were all capable of classifying AD samples and control samples with moderate accuracy (Figures 10C–E, 0.5 < AUC <0.7).

We further evaluated the expression levels of the six core GS&MetabolismRGs by utilizing the GSE97760 dataset as an external validation cohort (Figure 10F). The results revealed that four core GS&MetabolismRGs, *PTEN*, *AHR*, *NFKB1*, and *APP*, demonstrated extremely significant differences between AD and control samples (p < 0.001). Correlation analysis of the six core GS&MetabolismRGs in the GSE97760 dataset was performed (Figure 10G). Notably, *APP* and *PTEN* showed the strongest positive correlation (r = 0.79, p < 0.05), while *NFKB1* and *PTEN* exhibited the strongest negative correlation (r = −0.81, p < 0.05). Finally, ROC analysis (Figures 10H–J) demonstrated that the expression levels of the four core GS&MetabolismRGs, *NFKB1*, *AHR*, *PTEN*, and *APP*, were highly accurate in classifying AD samples and control samples (AUC >0.9). *TREM1* exhibited lower diagnostic accuracy, with an AUC ranging between 0.5 and 0.7.

### 3.10 Immune infiltration analysis in the disease and control group

**FIGURE 11:**
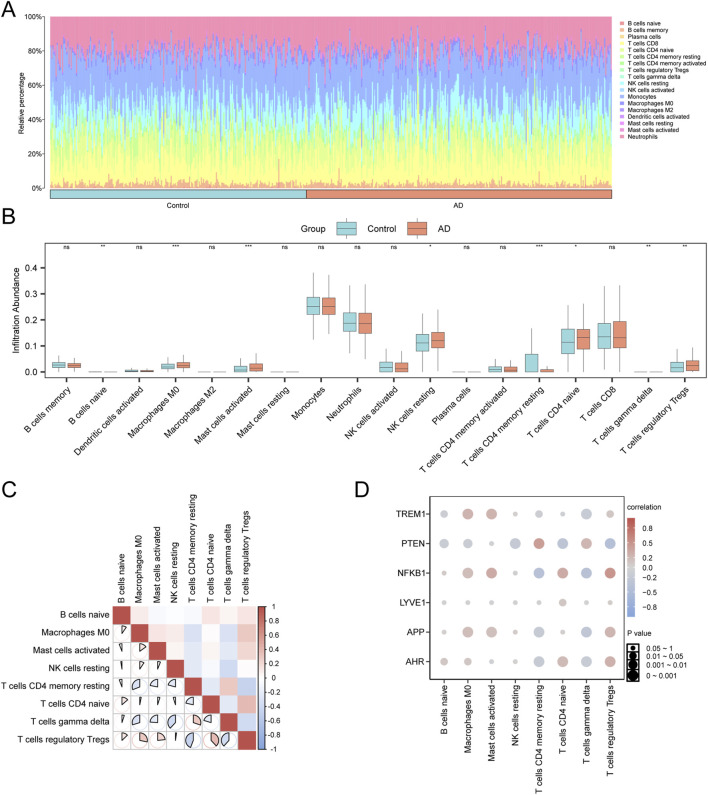
Immune infiltration analysis of combined datasets using the CIBERSORT algorithm A-B. The bar chart **(A)** and group comparison chart **(B)** show the proportion of immune cells in the integrated GEO dataset (combined dataset). **(C)** Heatmap of the immune cells in the integrated GEO dataset (combined datasets). **(D)** Bubble plot of the correlations between immune cell infiltration abundance and hub genes in the integrated GEO dataset (combined dataset). AD, Alzheimer’s disease; GEO, Gene Expression Omnibus. “ns” represents p-value ≥0.05 (not statistically significant), *p-value <0.05 (statistically significant), **p-value <0.01 (highly statistically significant), ***p-value <0.001 (extremely statistically significant). An absolute value of the correlation coefficient (r value) below 0.3 indicates weak or no correlation, between 0.3 and 0.5 indicates weak correlation, between 0.5 and 0.8 indicates moderate correlation, and above 0.8 indicates a strong correlation. Blue represents the control group and orange represents the AD group. Pink indicates positive correlations and blue indicates negative correlations. The depth of the color indicates the strength of the correlation.

To investigate the AD-associated immune microenvironment, the CIBERSORT algorithm was employed to evaluate the infiltration levels of immune cell types within the combined datasets. The results revealed significant differences in the infiltration levels of eight immune cell types (p < 0.05): resting memory CD4^+^ T, naïve B, naïve CD4^+^ T, M0 macrophages, regulatory T (Tregs), gamma delta T, resting NK, and activated mast cells (Figures 11A,B). Correlation analysis of immune cell infiltration in AD samples revealed that the majority of immune cells exhibited significant interrelationships. Notably, memory resting CD4^+^ T cells and Tregs demonstrated the strongest negative correlation (r = −0.441, p < 0.05) (Figure 11C). Furthermore, a correlation bubble plot revealed substantial interrelationships among most immune cell types, among which *NFKB1* showed a significant positive correlation with regulatory Tregs (r = 0.488, p < 0.05) (Figure 11D).

### 3.11 Immune infiltration analysis in the highRisk and lowRisk groups

**FIGURE 12:**
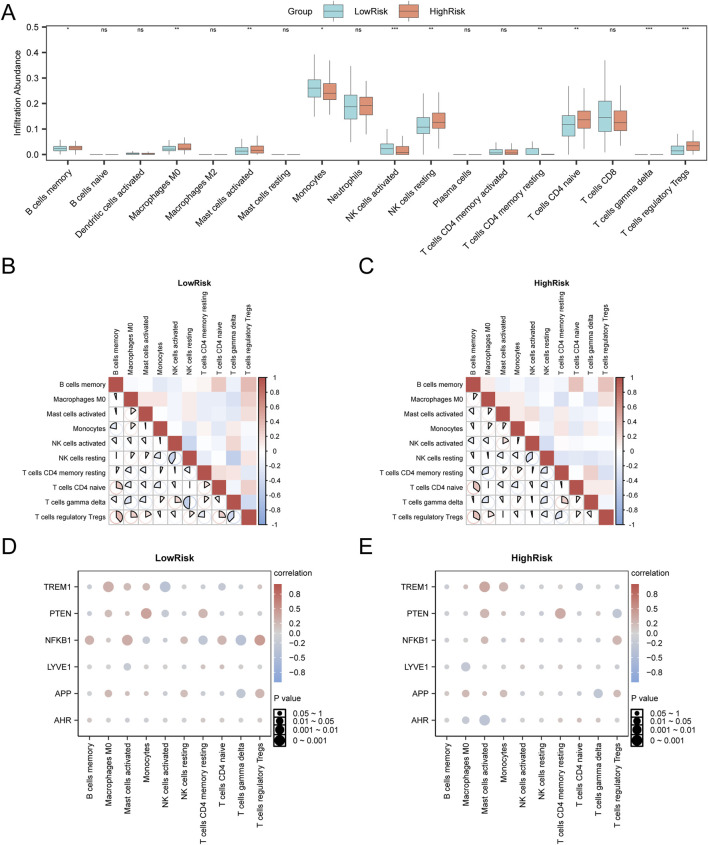
Immune infiltration analysis of risk groups using the CIBERSORT algorithm **(A)**. Grouping comparison diagram of immune cells in the low-risk (LowRisk) and high-risk (HighRisk) AD groups. **(B,C)**. Correlation heatmaps of immune cells in the low-risk (LowRisk) group **(B)** and high-risk (HighRisk) group **(C)** in AD samples. **(D,E)**. Bubble plots of correlations between the abundance of immune cell infiltration and hub genes in the low-risk (LowRisk) group **(D)** and high-risk (HighRisk) group **(E)** of AD samples. AD, Alzheimer’s disease. “ns” represents p-value ≥0.05 (not statistically significant), *p-value <0.05 (statistically significant), **p-value <0.01 (highly statistically significant), ***p-value <0.001 (extremely statistically significant). An absolute value of the correlation coefficient (r value) below 0.3 indicates weak or no correlation, between 0.3 and 0.5 indicates weak correlation, between 0.5 and 0.8 indicates moderate correlation, and above 0.8 indicates a strong correlation. Blue represents the LowRisk group and orange represents the HighRisk group. Pink indicates a positive correlation and blue indicates a negative correlation. The depth of the color indicates the strength of the correlation.

To investigate the potential involvement of the immune system in AD progression, we conducted an immune infiltration analysis comparing HighRisk and LowRisk AD groups. The results (Figure 12A) revealed statistically significant differences (p < 0.05) in the abundance of 10 immune cell types: Tregs, resting NK cells, memory B cells, naïve CD4^+^ T cells, activated NK cells, resting memory CD4^+^ T cells, gamma delta T cells, monocytes, M0 macrophages, and activated mast cells. Correlation analysis indicated that most immune cells showed a strong correlation in AD samples (Figures 12B,C). Notably, gamma delta T and resting NK cells exhibited the strongest negative correlation in LowRisk group (r = −0.485, p < 0.05). In contrast, in HighRisk group, memory B cells and naïve CD4^+^ T cells showed the strongest positive correlation (r = 0.355, p < 0.05) (Figure 12C). Correlation analysis (Figures 12D,E) further emphasized that *NFKB1* exhibited a significant positive correlation with Tregs in LowRisk group (r = 0.445, p < 0.05). In HighRisk group, *TREM1* expression exhibited a significant positive correlation with activated mast cells (r = 0.339, p < 0.05).

## 4 Discussion

AD is among the most common neurodegenerative disorders worldwide, characterized by the pathological accumulation of Aβ and tau protein, resulting in the progressive impairment of cognitive function (Shi et al., 2024). With the global population aging, the incidence of AD has increased, posing an increasing public health challenge. Recent studies have emphasized the pivotal role of GS in clearing Aβ and tau proteins. GS dysfunction exacerbates the accumulation of pathological proteins and neuroinflammation in AD. The complex interaction between metabolic status, immune system, and GS function is pivotal in AD pathogenesis. In this study, we integrated multiple datasets, including those from GEO and GeneCards, and conducted extensive bioinformatics analysis, ultimately identifying six GS&MetabolismRDEGs. We subsequently constructed and validated a novel diagnostic model for AD based on these core GS&MetabolismRGs to reveal their molecular characteristics and mechanisms in AD.

Initially, we identified six GS&MetabolismRDEGs, *NFKB1*, *AHR*, *PTEN*, *TREM1*, *APP*, and *LYVE1*, which were identified as crucial contributors to AD pathogenesis and progression. Logistic regression, SVM, LASSO shrinkage, and selection operator regression analyses confirmed that these genes are hub genes that play critical roles in AD. While genes such as *APP*, *PTEN*, and *NFKB1* are well-established in AD pathogenesis, the novelty of our study lies in their incorporation into a distinct GS&MetabolismRDEGs network, uncovering their synergistic interactions associated with GS dysfunction and metabolic regulation. Previous studies have highlighted the roles of *NFKB1* and *PTEN* in regulating neuroinflammation and apoptosis, which are involved in neurodegenerative diseases (Singh et al., 2020; Braun and Puglielli, 2022). *TREM1* activates downstream signaling cascades via the modulation of spleen tyrosine kinases (SYK), inducing neuroinflammation, a key driver of AD pathology (Anwar, 2023). Karam et al. previously identified *LYVE1* as a marker expressed by perivascular macrophages (PVMs), where it plays crucial roles in lymphatic drainage and immune function (Karam et al., 2022). Furthermore, AHR, the expressed protein, disrupts the GS through its interaction with aryl hydrocarbon receptor nuclear translocator–like protein 1 (BMAL1), thereby impairing the clearance of Aβ and tau proteins and worsening AD pathology (Salminen, 2023). Based on the above findings, we propose that these key genes synergistically initiate and accelerate AD by regulating glymphatic system architecture and function, modulating neuroinflammatory pathways, and altering amyloid-β and tau clearance efficiency. Future studies could utilize *in vivo* gene-edited mouse models, integrating fluorescent tracer assays with cerebrospinal fluid flow imaging, to directly assess how key genes influence glymphatic architecture and regulate amyloid-β and tau clearance. *In vitro* models, such as human brain organoids or glial-neuronal co-cultures, could elucidate the molecular pathways governing neuroinflammatory responses and metabolic regulation mediated by these genes. Integrated transcriptomic, proteomic, and metabolomic analyses would comprehensively delineate the resultant disruptions in signaling networks and their correlations with clinical phenotypes. Collectively, this approach would establish a robust theoretical and experimental framework for clarifying gene functions in the GS and AD, thereby identifying novel therapeutic targets.

Next, we performed functional enrichment analyses on the six identified GS&MetabolismRDEGs to identify the key MFs and biological pathways implicated in AD. Overall, the results revealed significant enrichment in several BP and signaling pathways, notably the hyaluronan metabolic process, PD-L1 expression, PD-1 checkpoint pathway in cancer. Hyaluronan (HA) is crucial for the formation and maintenance of the extracellular matrix, and is involved in the regulation of cell development, neuroplasticity, neurite outgrowth, and neuroinflammation (Sethi and Zaia, 2017; Kobayashi et al., 2020). Hyaluronan metabolism is activated in AD, resulting in abnormal extracellular matrix accumulation and enhanced neuroinflammation (Zakusilo et al., 2021). Targeting hyaluronan metabolism may help to restore normal extracellular matrix function and reduce neuroinflammation, thereby offering a potential therapeutic strategy for AD (Kobayashi et al., 2020). The PD-L1/PD-1 checkpoint pathway, which is traditionally known for its role in immune evasion in cancer, has garnered attention in the context of neurodegenerative diseases. Zhao et al. previously reported that the PD-L1/PD-1 pathway modulates hippocampal neuronal excitability and learning and memory behaviors (Zhao et al., 2023), whereas its dysregulation promotes Aβ plaque deposition in AD models (Topalian et al., 2012; Kummer et al., 2021). The success of PD-L1/PD-1 checkpoint inhibitors in cancer treatment, indicates their potential application in the treatment of neurodegenerative diseases (Topalian et al., 2012). The findings revealed that the core GS&MetabolismRDEGs synergistically drive AD pathogenesis through mechanisms involving immune-inflammatory modulation, extracellular matrix remodeling, and oxidative stress regulation, suggesting their potential as promising therapeutic targets.

Recent studies have highlighted the critical role of the GS in the clearance of metabolic waste and the maintenance of cerebral homeostasis (Doroszkiewicz et al., 2024; Perluigi et al., 2024). GS dysfunction exacerbates the accumulation of pathological proteins and neuroinflammation, and is closely associated with metabolic dysregulation and alterations in the immune microenvironment, thereby influencing the onset, progression, and clinical trajectory of AD. In contrast to single-pathway models, this study represents a pioneering effort to integrate glymphatic system- and metabolism-related genes to comprehensively capture the multifaceted, multi-pathway pathogenesis of AD. Through multi-omics analysis, we have developed a novel multi-gene diagnostic model for AD based on six core GS&MetabolismRDEGs, which exhibits significant advantages biological plausibility and clinical translational potential. Among the six core GS&MetabolismRDEGs, *LYVE1*, *AHR* and *TREM1* are emerging regulators of glymphatic function and metabolic homeostasis, offering a new perspective on AD inflammatory pathology. In the external validation set, *NFKB1*, *AHR*, *PTEN*, and *APP* show high accuracy, indicating excellent predictive power. The diagnostic model presented here relies on peripheral blood specimens and affords noninvasive sampling, operational simplicity and longitudinal monitoring, rendering it highly amenable to clinical translation. In contrast to traditional cerebrospinal fluid analysis or imaging-based diagnostics for AD, a peripheral blood transcriptomic assay offers significant advantages and, once broadly adopted, could enhance early detection and support risk-stratified management. However, several challenges must be addressed. First, peripheral blood exhibits considerable heterogeneity driven by age, comorbidities and individual immune status; therefore, the assay’s robustness and generalizability must be rigorously validated in large multicenter cohorts. Second, stringent standardization of sample processing and quality control procedures is essential to ensure reproducibility. Third, the relatively small sample size of the external validation cohort may limit the model’s generalizability and broader applicability. Due to current logistical constraints, the availability of external independent validation samples remains limited. Future studies aim to incorporate larger external multicenter datasets to further improve the model’s robustness and wider applicability. Finally, cost effectiveness, ease of implementation and ethical considerations will be critical for adoption in routine practice. Prospective multicenter studies that integrate multi-omics data with machine-learning methods will be critical to refine the model’s predictive power. Integration of multi-omics datasets with systematic immune microenvironment profiling has revealed the utility of molecular subtyping and early diagnosis, establishing a basis for precision stratification and targeted intervention in AD.

We further stratified the AD samples into HighScore and LowScore groups to explore the key gene sets and pathways associated with AD. GSEA of AD samples identified key pathways related to immune regulation, vascular dysfunction, transcriptional control, and inflammatory signaling, thereby highlighting the role of integrated multi-gene networks in the pathogenesis and progression of AD. The mRNA-TF and mRNA-miRNA regulatory networks revealed that TF (SPI1) and miRNAs (*miR-520c-3p*) were critical regulators of hub genes, exerting pivotal roles at the transcriptional and post-transcriptional levels, respectively. *SPI1* further modulates microglial phagocytosis and immune responses, while regulating the expression of both proinflammatory and lipid metabolism-related genes implicated in AD (Rustenhoven et al., 2018; Kim et al., 2024). Previous studies have revealed that silencing *SPI1* exacerbates Aβ accumulation and plaque deposition, thereby accelerating AD progression (Kim et al., 2024). These findings shed light on the novel molecular mechanisms driving the pathogenesis of AD and provide a foundation for identifying potential therapeutic targets.

Finally, we conducted immune infiltration analysis in both the disease control and HighRisk and LowRisk groups. The results revealed significant changes in the infiltration of various immune cell types in AD samples, with Tregs and activated mast cells exhibiting strong correlations with core GS&MetabolismRDEGs, such as *NFKB1* and *TREM1*. Tregs are crucial for immune tolerance and homeostasis, and their dysfunction occurs early in neurodegenerative diseases (He and Balling, 2013). In the present study, we identified a strong correlation between Treg infiltration in the LowRisk group and AD, indicating that Tregs exert immunosuppressive effects during the early stages of AD by mitigating neuroinflammation. Conversely, in the HighRisk AD group, a more pronounced immune response was observed, with activated mast cells likely contributing to neuroinflammation and disease progression (Wilson et al., 2024). Overall, these findings highlight the vital role of immune cells in modulating neuroinflammation and driving AD progression. The observed correlations among immune cells in this study indicate promising therapeutic strategies. For instance, modulating Treg activity during the early stages of AD may slow disease progression, while targeting mast cell activation in advanced stages of AD could help reduce neuroinflammation, thereby supporting the development of personalized interventions for specific AD stages. Monitoring Treg and mast cell activity could further guide the development of immune-based biomarkers for early AD detection and risk stratification, facilitating timely interventions. Future experimental studies exploring these immune cells as potential therapeutic targets could enhance the clinical management of AD and advance the development of personalized immunomodulatory therapies.

This study had several limitations. First, the lack of wet-lab experimental validation may limit the translational applicability of our findings. Future studies should employ both *in vitro* and *in vivo* models to explore the functional roles and underlying molecular mechanisms of key genes, such as *NFKB1* and *AHR*, and assess the potential for early intervention and stratification strategies guided by GS&MetabolismRDEGs. Second, the relatively small sample size may introduce statistical bias and limit the generalizability of the findings. To further enhance the model, future multicenter studies involving larger and more diverse patient cohorts are essential to validate the robustness and clinical relevance of the identified biomarkers. Furthermore, sensitivity analysis methods, such as bootstrap resampling, data down-sampling, and cross-validation, will be adopted to systematically evaluate the stability of diagnostic models under different sample sizes and distributions. Third, the lack of longitudinal follow-up data restricts the ability to assess disease progression and the prognostic value of the identified biomarkers. Incorporating longitudinal designs in future research may provide deeper insights into temporal dynamics. Moreover, gender- and age-specific analyses were not performed, despite well-documented differences in AD susceptibility and progression. Future studies should include such stratifications to improve clinical relevance and facilitate personalized applications.

## 5 Conclusion

This study successfully identified six GS&MetabolismRDEGs associated with AD, and subsequently constructed a novel diagnostic model with excellent predictive performance. These findings deepen our understanding of the molecular mechanisms driving AD and highlight the potential roles of the GS and metabolic pathways in disease progression. These GS&MetabolismRDEGs hold significant promise as key biomarkers for the early diagnosis of AD.

## Data Availability

Publicly available datasets were analyzed in this study. This data can be found here: https://www.ncbi.nlm.nih.gov/geo/.
